# Localization of acoustic modes in periodic porous silicon structures

**DOI:** 10.1186/1556-276X-9-419

**Published:** 2014-08-21

**Authors:** Zorayda Lazcano, Octavio Meza, Jesús Arriaga

**Affiliations:** 1 Instituto de Física, Benemérita Universidad Autónoma de Puebla, 18 Sur y San Claudio, Edif. 110-A, Puebla 72570, México

**Keywords:** Porous silicon, Localized defects, Phononic crystals, Acoustic transmission

## Abstract

The propagation of longitudinal acoustic waves in multilayer structures based on porous silicon and the experimental measurement of acoustic transmission for the structures in the gigahertz range are reported and studied theoretically. The considered structures exhibit band gaps in the transmission spectrum and these are localized modes inside the band gap, coming from defect layers introduced in periodic systems. The frequency at which the acoustic resonances appear can be tuned by changing the porosity and/or thickness of the defect layer.

## Background

The study of acoustic and elastic wave propagation in phononic crystals (PCs) [1-3] have been studied theoretically [4] and experimentally [5] in recent years. In analogy with the photonic band gap materials, emphasis in phononic crystals has been on achieving large acoustic band gaps within which propagation of sound is forbidden.

The interest about phononic band gap materials is in both, pure and applied physics. Fundamental physics has a special interest concerned with the localization phenomena of sound and vibrations in PCs. Researchers have prospected numerous applications based on cavity structures built around PCs, such as wave filters, waveguides, and splitters [6-9]. Furthermore, it is possible to design cavities for coherent (single-wavelength) phonon generation and control, to attain phonon amplification and ‘lasing’ in the called ‘saser’, one of the most important potential applications [10-12].

Periodic solid-state structures exhibit transmission stop bands for waves at certain frequencies. By placing one or more defects into a perfect phononic crystal, acoustic cavities are created inside the system. The presence of these defects, produces localization of elastic or acoustic modes inside the phononic band gap. These localized modes are the acoustic analog of donor or acceptor states produced inside the band gap of semiconductors. In analogy with electronic systems, one can consider these acoustic states to control the sound propagation through the structure.

If a defect is introduced into a periodic structure, the translational symmetry is broken and highly localized defect modes within the band gaps are created [6,8,13,14]. Point, linear, and planar defect states have been theoretically investigated in one-dimensional (1D), two-dimensional (2D), and three-dimensional (3D) phononic crystals [3,15,16]. In 1D structures, a microcavity can be a spacer layer of thickness *λ*/2 enclosed by two Bragg reflectors [17]. In 2002, Trigo et al. proposed phonon cavities in structures consisting of two semiconductor superlattices enclosing a spacer layer, showing that acoustical phonons can be confined in such layered structures if the spacer thickness is an integer multiple of the acoustic half-wavelength at the center of one of the superlattice-folded minigaps. These acoustic cavities are semiconductor multilayers in the nanometer scale and are fabricated by molecular beam epitaxy (MBE), which is a sophisticated and expensive technique that requires ultra-high vacuum system and a very tight control on the growth parameters, and modulate the thicknesses is easier than to modulate the elastic properties of the layers. Contrasting, porous silicon (PS) multilayer fabrication is relatively easy and considerably less costly, besides that this material allows to modulate both the thicknesses and the elastic properties of each layer.

PS is known as a versatile material with applications in light emission, sensing, and photonic devices [18]. The possibility of producing acoustic band gaps in PS was proposed in 2005 [19], and detailed calculations of predicted bandwidths were subsequently published [20]. Recently, experimental results of Brillouin light scattering suggested the existence of zone-folded phonons and phononic band gaps in PS multilayers [21]. G. N. Aliev et al. in 2010 reported experimental measurements of transmission for longitudinal acoustic waves and observation of hypersonic band gaps in a periodic PS structure with an acoustic microcavity consisting of a spacer layer of thickness *λ*/2 [22].

By taking advantage of the possibility to modulate the elastic properties of PS layers, and considering that it is possible to create localized modes by introducing a defect layer with different acoustic properties into a periodic structure, in this paper, we investigate the propagation of longitudinal acoustic waves in multilayer structures based on PS, that exhibit resonant cavity modes in frequencies of gigahertz (GHz), consisting of defect layers intentionally introduced in periodic structures. The design and material parameters that allow to create these localized acoustic modes is discussed, and experimental results of the measured acoustic transmission in PS samples fabricated by electrochemical etching are presented.

## Methods

### Theoretical models

The multilayer PS structures studied here have thicknesses in micrometer range and the procedure used to fabricate them creates mesoporous silicon with an average pore diameter of 20 to 50 nm. On the other hand, in our experiments, the typical longitudinal wavelengths excited throughout the samples are 3 to 7 *μ*m depending on porosity. Accordingly, each of the individual layers in the structures is assumed to be homogeneous.

The longitudinal acoustic wave equation in the continuum limit for a solid inhomogeneous along the *z* direction (but homogeneous along the *x* and *y* directions) is given by [23],

(1) $$\rho_{j}\frac{\partial^{2}u(z,t)}{\partial t^{2}}={\left(\lambda +2\mu \right)}_{j}\frac{\partial^{2}u(z,t)}{\partial z^{2}},$$

where *ρ*_*j*_ is the mass density, ${\left(\lambda +2\mu \right)}_{j}=\rho v_{\text{Lj}}^{2}$ and *u*(*z*,*t*) is the atomic displacement. Here, *j* is an index identifying each layer. The limits of the elastic continuum description of wave propagation in ordered media depends on the dimensions of the system compared with the wavelength. When the dimensions approach nanometer-length scales, atomistic treatments using first principles or semi-empirical methods may become necessary [24]. However, in our case, the thicknesses of the layers are in the micrometer range and each layer can be considered as a homogeneous layer; thus, the model described before is assumed valid.

In a solid, the acoustic waves can be longitudinal or transversal. In this letter, only longitudinal waves propagating through PS are considered because in our experiments, the waves are coupled to the samples through a liquid at normal incidence. The mass density *ρ* is a function of the porosity and is described by *ρ*=*ρ*_0_(1−*P*) where *ρ*_0_=2.330 g/cm ^3^ is the density of bulk silicon and *P* the porosity. The acoustic velocity dependence on porosity is given empirically by *v*_*L*_=*v*_*L*0_(1−*P*)^*k*^, being *v*_*L*0_ the longitudinal velocity of sound in bulk silicon along the (100) crystallographic direction and *k*≥0.5 is a constant [25-28]. In general, the parameter *k* depends on PS morphology which in turn depends on the doping level of the Si substrate [25,26].

The general solution of Equation 1 takes the form,

(2) $$u(z_{j},t)=\left[A_{j}^{+}\exp\left({\text{ik}}_{j}z_{j}\right)+A_{j}^{-}\exp\left(-{\text{ik}}_{j}z_{j}\right)\right]\exp(\mathrm{i\omega t}),$$

corresponding to waves propagating to the right (*A*^+^) and left (*A*^−^). At each interface, this solution must satisfy the boundary conditions related to the continuity of the atomic displacement and stress,

(3) $$u_{j}\left(d_{j}\right)=u_{j+1}\left(d_{j}\right)$$

and

(4) $$\rho_{j}v_{\text{Lj}}^{2}\frac{\partial u_{j}\left(d_{j}\right)}{\mathrm{\partial z}}{\left|{\;}_{{z=d}_{j}}=\rho_{j+1}v_{L(j+1)}^{2}\frac{\partial u_{j+1}\left(d_{j}\right)}{\mathrm{\partial z}}\right|}_{{z=d}_{j}}$$

respectively. Here, *d*_*j*_ denotes the position of the *j*-th interface between *j* and *j*+1 layers. The frequency *ω* is related to its wave vector via *ω*=*k*_*j*_*v*_*j*_, with *v*_*j*_ the sound speed in the *j*-th layer and *ω*=2*πf*, being *f* is the frequency in *s*^−1^. Using the transfer matrix method (TMM) [26], we can relate the amplitudes of the fields $A_{j}^{+}$ and $A_{j}^{-}$ in the layer *j* of the system with the amplitudes of the wave in the *j*+1 layer according to

(5) $$\left(\begin{array}{c} A_{j}^{+}\\ A_{j}^{-} \end{array}\right)=T_{j}\left(\begin{array}{c} A_{j+1}^{+}\\ A_{j+1}^{-} \end{array}\right).$$

The transfer matrix *T*_*j*_ appearing in the previous equation propagates the amplitudes through a layer with thickness *d*_*j*_, mass density *ρ*_*j*_, and sound longitudinal velocity *v*_*Lj*_, and is given explicitly by,

(6) $$T_{j}=\left(\begin{array}{cc} \cos\left(k_{j}d_{j}\right) & \frac{-i\sin\left(k_{j}d_{j}\right)}{k_{j}v_{\text{Lj}}^{2}\rho_{j}}\\ -{\text{ik}}_{j}v_{\text{Lj}}^{2}\rho_{j}\sin\left(k_{j}d_{j}\right) & \cos\left(k_{j}d_{j}\right) \end{array}\right).$$

If we consider a structure formed by *N* layers, the total transfer matrix representing the structure is obtained by multiplying, in the appropriate order, a series of *N* transfer matrices, each one given by a matrix of the type appearing in Equation 6. The obtained matrix relates the displacement vector at the beginning of the structure with that at the end, and represents a 2 × 2 set of equations that can be fully solved.

With the above formalism, one can derive the acoustic eigenenergies and eigenvectors. The reflectivity and transmission can also be calculated as the square modulus of $A_{0}^{-}$ and $A_{N}^{+}$, respectively, by imposing the boundary conditions $A_{0}^{+}=1$ and $A_{N}^{-}=0$ for a wave traveling from right to left. Here 0 and *N* label the first and last layer of the structure, respectively.

Attenuation can be included by taking the wave vector *k*_*j*_ complex, such that *K*_*j*_=*k*_*j*_−*α*_*i*_, where *α*_*i*_ is attenuation coefficient. The form of the attenuation coefficient depends on the physical process causing loss and we assume that the Akhiezer model is dominant in a semiconducting material. This gives *α*=*ηω*^2^/2*ρv*^3^, where *η* is the viscosity. However, it is known that introducing acoustic attenuation into the model leads to important effects as the shrinking of gaps, only for frequencies higher than 180 GHz [29]; therefore, no absorptive behavior is considered in our model since no important effects are obtained if they are included.

Furthermore, the position and width of the band gap are critical parameters for devices that reflect or localize the acoustic waves [30]. Band structures of many kinds of periodic phononic crystals have been reported [31-33]. The most commonly studied acoustic band gaps in 1D PCs are the Bragg type, appearing at an angular frequency *ω* of the order of *v*_*L*(*T*)_/*d* (*v*_*L*(*T*)_ refers to the longitudinal (transverse) elastic wave velocity and *d* is the lattice constant).

An acoustic Bragg mirror can be made by repeating *n* times a basic block of two materials with different acoustic properties. The mismatch in the acoustic impedance between these layers results in waves that are reflected and interfere, giving an acoustic band gap around a central frequency *f*_*B*_. For normal incidence, this frequency is given by

(7) $$f_{B}=\frac{2}{m}{\left(\rho_{1}d_{1}/Z_{1}+\rho_{2}d_{2}/Z_{2}\right)}^{-1},\;\;\;\;m=1,2,3,\ldots ,$$

being *m* the order of the stop band, *d*_1_ and *d*_2_ are the layer thicknesses, and *Z*_1_ and *Z*_2_ are the acoustic impedances of layers 1 and 2, respectively. The acoustic impedance *Z* is given by *ρv*, with *v* as the sound velocity and *ρ* as the mass density. The condition *ρ*_1_*d*_1_/*Z*_1_=*ρ*_2_*d*_2_/3*Z*_2_ optimize the stop-band width and reflectivity, corresponding in an infinite stack, to the first minigap at the Brillouin zone center. The reflectivity at the center of the stop-band depends on the acoustic impedance mismatch between the two materials *Z*_2_/*Z*_1_, and for *n* pairs of layers is given by [17,22],

(8) $$R_{B}={\left[\frac{{\left(Z_{2}/Z_{1}\right)}^{2n}-1}{{\left(Z_{2}/Z_{1}\right)}^{2n}+1}\right]}^{2}.$$

In [34], the authors considered periodic semiconductor structures of GaAs/AlAs to introduce microcavities as spacer layers of thickness *λ*/2. However, for a 10-period GaAs/AlAs mirror, *R*_*B*_∼0.880, while *R*_*B*_∼0.996 if *n*=20. For a PS structure, a porosity variation of 15 *%* between the constituent layers of 52 *%* and 67 *%* of porosity, leads to *R*_*B*_∼0.997 for *n*=6. Thus, by modulating the porosity of the PS structures, very high reflectivity values can be achieved. This is an essential condition to obtain narrow transmission bands into the stop bands corresponding to the cavity modes.

To demonstrate the localization in time domain, we consider the propagation of a Gaussian pulse through the structure. The Gaussian pulse is described by *g*(*f*)= exp(−4*π*[(*f*−*f*_0_)/*σ*]^2^), were *f*_0_ is the central frequency and *σ* the pulse width. In response to the incident pulse, the time and spatial variations of the displacement field *u*(*z*,*t*) inside the sample can be calculated according to the scattering state method as [35],

(9) $$u(z,t)=\frac{1}{2\pi }\int_{-\infty }^{+\infty }u(z,f)g(f)e^{-i2\mathrm{\pi ft}}\text{df}$$

where *u*(*z*,*f*) is the displacement field distribution at each frequency, which is obtained by the transfer matrix method.

### Experimental details

Samples were electrochemically etched from boron-doped (100)-oriented Si substrates with a resistivity of 0.007 to 0.013 *Ω*cm. Room-temperature anodization was performed using a 1:1 solution of HF (40 *%*) and ethanol (99.98 *%*). The acoustic transmission measurements reported here were done using a Vector Network Analyzer (VNA). Each sample was placed between two ZnO-based piezoelectric transducers with a central frequency of 1.1 GHz and an operation bandwidth of 500 MHz. The transducers consist of a piezoelectric layer driving waves into a silicon pillar with a thickness of 520 *μ*m. To couple the transducers to the specimen, In-Ga eutectic was used. The transducer front surface was aligned parallel to the sample surface using two orthogonal microscopes so that the acoustic waves impinge normally into the PS layers. The transducers were connected to the VNA ports and transmission parameters were measured as function of frequency, more details of the experimental set-up can be found in [36]. Dependence of sound velocity with porosity for Si wafers and experimental conditions used here were determined previously and reported in [36], the values of *v*_*L*0_ and the parameter *k* used here are 8.44 km/s and 0.56, respectively.

For the fabrication of PS multilayers, we consider the inclusion of ‘etch stops’ or ‘etch breaks’ where the current is interrupted to stop the etching of the Si wafer in order to prevent any depletion of HF [37]. The introduction of these etching breaks is necessary to obtain layers with constant porosity with depth [38]. Because our samples include very thick layers, with large mismatch porosities between them, the number and length of the etch breaks are important to obtain homogeneous structures. We found that etch breaks of 4 s with a ratio (etch break time)/(etching time) from 3.3 for low porosities (52 *%*) to 7.3 for high porosities (67 *%*) are enough to minimize any chirp in the layers.

## Results and discussion

Thicknesses of layers were measured by optical microscopy, and the layer porosities were determined from optical reflectance spectra by fitting our experimental measurements and comparing them with our theoretical simulations for each sample. The acoustic transmission and field intensity distribution have been modeled using the transfer matrix method described before and taking into account the effect of the sample (PS-Si substrate), transducers (Si pillars), and In-Ga eutectic liquid used to couple the transducers to the sample.

**Figure 1:**
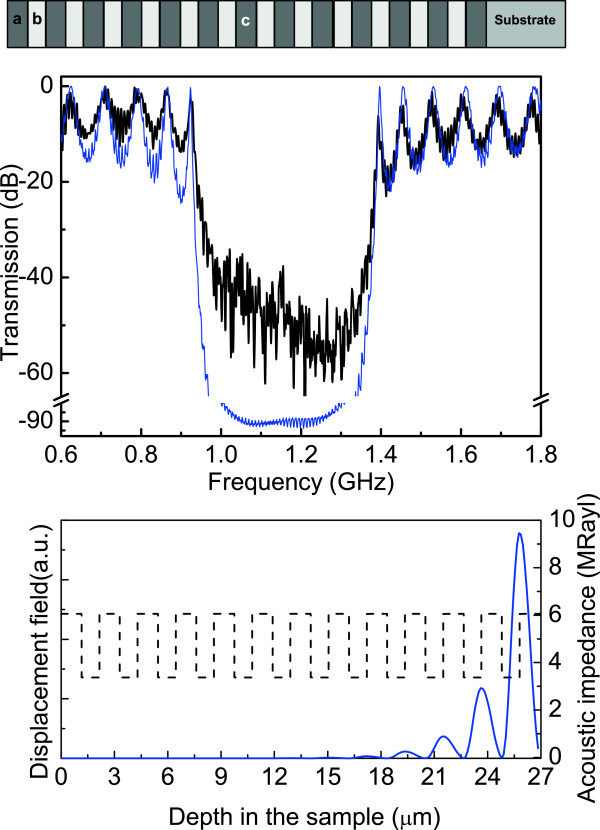
**Acoustic transmission and distribution of the displacement field for the periodic case, sample 1.** (Top) Scheme of the periodic structure consisting of 12.5 periods of layers *a* and *b*. (Middle) Acoustic transmission spectra, measured in solid line and calculated in dashed line. The measured transmission, recorded on a logarithmic scale, is normalized to its maximum and corrected by an envelope function of the transducer response. (Bottom) In solid line, squared phonon displacement corresponding to the central frequency of the gap. The dashed line represents the material acoustic impedance and serve to identify the position in the sample.

Three PS multilayer samples are considered here to show the effect of localization inside the structures. All of them consist of layers *a* and *b* repeating alternatively, and a defect layer, *c*, in the middle of the structure. The sequence used for structures was *abababababab*−*c*−*babababababa*=(*ab*)^6^*c*(*ba*)^6^. In the first sample (1) porosities and thicknesses of layers *a* and *b* are *P*_*a*_=53*%*, *d*_*a*_=1.15 *μ*m, *P*_*b*_=67*%*, and *d*_*b*_=1.10 *μ*m, respectively. Here, layer *c* has the same thickness and porosity of layer *a*, and therefore, this sample is completely periodic. The porosities and thicknesses of the layers were chosen to obtain the fundamental stop band within the bandwidth of the acoustic transducers, and satisfying Equation 7. A scheme of structure 1 is displayed in the top panel of Figure 1. The central panel of Figure 1 (solid line) shows the measured acoustic transmission spectrum of the PS periodic structure with a total thickness of approximately 27 *μ*m. The band gap in the transmission spectrum observed around 1.15 GHz and ranged from 0.94 to 1.38 GHz is the first-order acoustic stop band of the mirror, corresponding to *m*=1 in Equation 7. This fundamental stop band shows an attenuation of approximately 50 dB with a fractional bandwidth of 37 *%*. The dashed line is the result of calculations using TMM. Good agreement between modeled and measured spectra is seen. The fine features of the spectrum are not noise but the longitudinal modes of the Si pillars of the transducers and the Si substrate of the sample. The fundamental band gap has a depth of approximately 50 dB which is less than the modeled value of approximately 100 dB. However, this is an experimental limitation on the setup used and its noise floor. Thus, we can conclude that the stop band has a depth of at least 50 dB. The bottom panel of Figure 1 shows the squared displacement field corresponding to the central frequency of the gap, 1.15 GHz. The dashed line represents the material acoustic impedance and is useful to identify the position in the sample. As can be seen, the displacement field is not localized, as is expected.

Now, based on the concepts mentioned before about cavities, we will show how the intentional introduction of a defect layer between a pair of mirrors can lead to formation of an acoustic cavity mode within the stop band. For this purpose, we consider two structures: sample 2 and sample 3.

**Figure 2:**
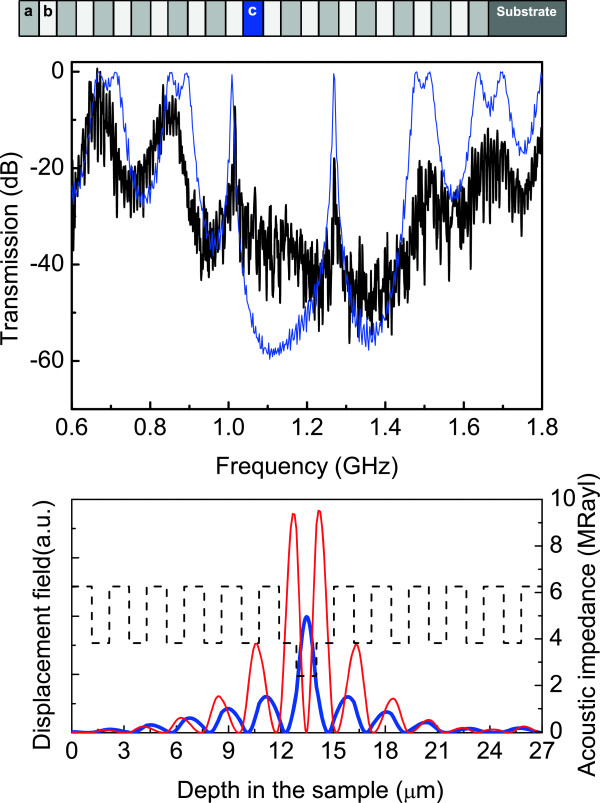
**Acoustic transmission and distribution of the displacement field for sample 2.** (Top) Scheme of a structure consisting of two mirrors with six periods of layers *a* and *b* enclosing a defect layer of higher porosity between them. (Middle) Measured acoustic wave transmission spectrum through the sample (solid line). The dashed curve is the calculated spectrum (see text for details). (Bottom) In solid line, squared phonon displacement corresponding to the first (thick line) and second (thin line) modes at 1.01 and 1.27 GHz, respectively. The dashed line represent the layer acoustic impedance.

In sample 2, porosities and thicknesses of layers *a*, *b*, and *c* are: *d*_*a*_=1.15 *μ*m, *P*_*a*_=52*%*, *d*_*b*_=1.00 *μ*m, *P*_*b*_=65*%*, *d*_*c*_=1.15 *μ*m and *P*_*c*_=74*%*, respectively. The defect (layer *c*) corresponds to a layer with the same thickness, as the periodic case, but higher porosity (lower impedance), as is shown schematically at the top of Figure 2. In the middle of Figure 2 are shown the acoustic transmission spectra, measured experimentally (solid line) and calculated theoretically (dashed line). The introduction of the defect layer results in well-localized transmission modes at 1.01 and 1.27 GHz, within the fundamental stop band ranged from 1.02 to 1.47 GHz and with a fractional bandwidth of 35 *%*, as it can be seen in the transmission spectrum. At the bottom of the Figure 2 is shown (in solid line) the displacement field distribution as a function of the position in the sample, corresponding to the cavity modes, the first (thick line) and second (thin line) modes at 1.01 and 1.27 GHz, respectively. It can be seen that the amplitude of the acoustic displacement is maximum around the defect layer. The dashed line is the material acoustic impedance.

**Figure 3:**
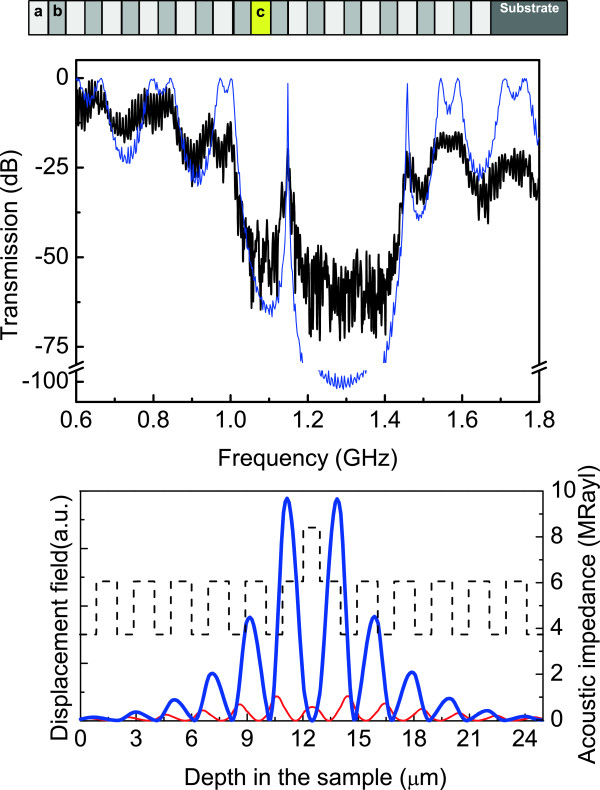
**Acoustic transmission and distribution of the displacement field for sample 3.** (Top) Scheme of a structure of two mirrors with six periods of layers *a* and *b* enclosing a defect layer of lower porosity. (Middle) Measured (solid line) and calculated acoustic transmission spectra (see text for details). (Bottom) In solid line, squared phonon displacement corresponding to the cavity mode frequency (thick line) at 1.15 GHz, and for a frequency of 1.46 GHz (thin line). The dashed line represent the layer acoustic impedance.

Sample 3, represented schematically at the top of Figure 3, contains a defect consisting of a layer with lower porosity (higher impedance) at the center of the structure. Here, thickness and porosities are: *d*_*a*_=0.89 *μ*m, *P*_*a*_=65.5*%*, *d*_*b*_=1.12 *μ*m, *P*_*b*_=53*%*, *d*_*c*_=0.89 *μ*m, *P*_*c*_=42*%*, for layers *a*, *b*, and *c*, respectively. The defect layer (*c*) keeps the periodicity in thickness but the porosity changes. As it can be clearly seen in measured transmission spectrum shown in Figure 3, this results in an acoustic cavity mode at 1.15 GHz within the fundamental stop band ranging from 1.02 to 1.44 GHz (34 *%* fractional bandwidth). The corresponding displacement field distribution for this cavity mode is shown at the bottom of the same figure (thick line) and demonstrates that the displacement field is maximum around this cavity in the same way as the second mode in sample 2. For demonstration purposes, we have calculated the displacement field for 1.46 GHz and the results are shown in Figure 3 using a thin line. Localization effects cannot be observed.In Figures 1, 2, and 3, good agreement between modeled and measured spectra is observed, and the slight differences between theoretical and experimental acoustic transmissions are due to features of porous silicon layers which are not considered here, as the roughness at the interfaces, as well as intrinsic error coming from the measured procedure, and not to absorption properties, as was explained before.

**Figure 4:**
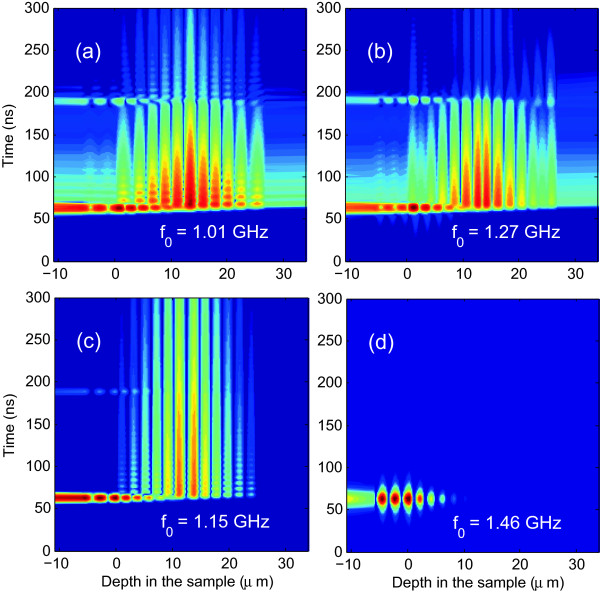
**Time evolution of acoustic Gaussian pulses.** Calculations of *ln*(*u*(*z*,*t*)) for acoustic Gaussian pulses centered at the frequency *f*_0_ indicated in each figure and *σ*=200 MHz for all cases. **(a**,**b)** Sample 2. **(c**,**d)** Sample 3. Zero in x-axis is placed at the surface of the PS sample.

In Figure 4, we show the time-resolved displacement field *u*(*z*,*t*), corresponding to the time evolution of a Gaussian pulse in the samples calculated using Equation 9. Figure 4a,b corresponds to the time and spatial variations of the displacement field inside sample 2, using *f*_0_=1.01 GHz in Figure 4a and 1.27 GHz in Figure 4b. These values correspond to the frequencies where the first and the second cavity modes appear, respectively. Figure 4c shows the displacement field inside of sample 3 for *f*_0_=1.15 GHz, the frequency of the corresponding cavity mode. Figure 4d corresponds to sample 3 using *f*_0_=1.46 GHz. We use a pulse with *σ*=200 MHz for all cases. In Figure 4a, it can be seen that the displacement field is in the center of the PS structure, corresponding to the defect layer. Figure 4b corresponds to the same structure but the incident Gaussian pulse has a frequency equal to 1.27 GHz; in this case, localization is around the defect layer and not inside it. In Figure 4c, as Figure 4b, the localization is around the defect layer and not inside it, because this corresponds to the second mode of sample 3. Calculations for sample 3 for the peak at 1.46 GHz appears in Figure 4d, as can be seen, localization of the displacement field is not observed.

**Figure 5:**
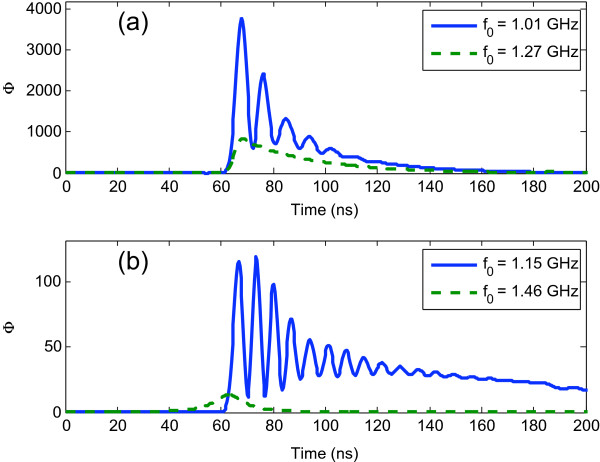
**Displacement field intensity as a function of time.** Theoretical calculations for the displacement field intensity *u*(*z*,*t*)^2^ in the defect as a function of time for **(a)** sample 2 and **(b)** sample 3, for frequencies indicated in each figure.

In order to estimate the displacement field intensity *u*(*z*,*t*)^2^, within the defect layer as a function to the time, we integrate the displacement field on the defect layer using,

(10) $$\Phi (t)=\int_{\text{defect}}u^{2}(z,t)\text{dz},$$

where *Φ*(*t*) is the displacement field intensity contained in the defect as a function of the time. Figure 5a shows *Φ*(*t*) for sample 2 for two Gaussian pulses centered in the frequencies *f*_0_ indicated there, as expected the first mode has higher displacement field intensity in the defect layer because the acoustic wave is localized in the center of the PS structure, on the contrary to the second mode, see Figure 5a. In the case of sample 3, the localization is less than sample 2 for the two Gaussian pulses considered, that is, the *Φ*(*t*) amplitude for sample 2, see Figure 5b, in the first mode is around 30 times more the *Φ*(*t*) amplitude in sample 3 for one incident Gaussian pulse with frequency equal to 1.15 GHz. Finally, localization is not observed in sample 3 for a Gaussian pulse with a frequency of 1.46 GHz, as is expected, see Figure 5b.

The modeled transmittance of the periodic case (sample 1) and for the two cavity structures (samples 2 and 3), obtained by the TMM, shows a good match with the experimental results. The localized acoustic resonances can be tuned at different frequencies (within the acoustic band gap) by changing the porosity of the defect layer.

Moreover, for commercial acoustic mirrors which are components of solidly mounted resonators and filters [39], a low-acoustic-impedance material such as SiO _2_ is layered with high-impedance materials such as tungsten or molybdenum. Following Equation 8, for the layer pair of molybdenum and silica, where acoustic impedances are 66.2 MRayl and 13.1 MRayl, respectively, the fixed impedance ratio is 5.1, and the same impedance ratio can be obtained using PS layers of 30 *%* and 75 *%*, so, by modulating the porosity, very high reflectivity values can be achieved. Besides, by increasing the number of pairs of layers enclosing the defect layer leads to narrowing of the transmission band together with deepening of the stop band.

## Conclusions

We have demonstrated theoretically by using the TMM and experimentally by acoustic transmission measured directly, the formation of acoustic cavity modes in GHz frequencies by introduction of defects into periodic structures based on PS. Acoustic resonances can be tuned at different frequencies by changing the porosity of the defect. And we proved that these resonant modes appear due to the localization of the field into the defect. The acoustic mirrors and cavity structures based on PS have a performance which is at least comparable with that devices based on semiconductor superlattices. This study could be useful for the design of acoustic devices, such as highly selective frequency filters with applications in GHz range.

## Competing interests

The authors declare that they have no competing interests.

## Authors’ contributions

JA had the original idea of the study. ZL performed the experiments and measurements. OM and ZL did the numerical calculations. All authors contributed in the writing of the manuscript. All authors read and approved the final manuscript.
